# Social preferences and cognitive reflection: evidence from a dictator game experiment

**DOI:** 10.3389/fnbeh.2015.00146

**Published:** 2015-06-19

**Authors:** Giovanni Ponti, Ismael Rodriguez-Lara

**Affiliations:** ^1^Laboratory of Theoretical and Experimental Economics (LaTEx), Departamento de Fundamentos del Análisis Económico, Universidad de AlicanteAlicante, Spain; ^2^Dipartimento di Economia e Finanza, LUISS Guido CarliRoma, Italy; ^3^Department of Economics, Middlesex University LondonLondon, UK

**Keywords:** cognitive reflection, social preferences, experimental economics, behavioral economics, dictator games

## Abstract

This paper provides experimental evidence on the relationship between social preferences and cognitive abilities, which we measure using the Cognitive Reflection Test (CRT). We elicit social preferences by way of 24 dictatorial situations, in which the Dictator's choice sets include (*i*) *standard* Dictator games, where increasing the Dictator's payoff yields a loss for the Recipient, (*ii*) *efficient* Dictator games, where increasing the Dictator's payoff also increases that the Recipient's; as well as other situations in which (*iii*) either the Dictator's or (*iv*) the Recipient's monetary payoff is held constant. We partition our subject pool into three groups: *reflective* (scoring 2 or more in the CRT)*, impulsive* (opting twice or more for the “intuitive” but wrong answers in the CRT) and the remainder. We find that impulsive Dictators show a marked *inequity aversion attitude*, especially in standard Dictator Games. By contrast, reflective Dictators show lower distributional concerns, except for the situations in which the Dictators' payoff is held constant. In this case, reflective Dictators give significantly more.

## Introduction

Researchers have made substantial improvements in understanding the relationship between various measures of cognitive ability and economic behavior in different domains. In this respect, measures of cognitive ability have been shown to determine the degree of strategic sophistication (e.g., Rydval et al., 2009; Brañas-Garza et al., 2012; Carpenter et al., 2013) and appear to correlate with risk and time preferences (Frederick, 2005; Brañas-Garza et al., 2008; Burks et al., 2009; Dohmen et al., 2010; Andersson et al., 2013; Benjamin et al., 2013), as well as with heuristics and well-known behavioral biases in financial decisions, such as *overconfidence, anchoring* or the so-called *conjunction fallacy* (Oechssler et al., 2009; Bergman et al., 2010; Hoppe and Kusterer, 2011; Toplak et al., 2011).

Despite that there have been noteworthy advances in the literature on pro-social behavior over the last years, the relation between social preferences and cognitive abilities is still sparse and far from univocal. Chen et al. (2013) find that subjects who perform better in the Math portion of the SAT (formerly referred as the Scholastic Aptitude Test) are more generous in both the Dictator Game and in a series of small-stakes “dictatorial” (i.e., unilateral) decisions, known as *Social Value Orientation* (SVO), albeit subjects with higher Grade Point Average (GPA) outcomes tend to be more selfish in dictator decisions. This latter result is in line with those of Ben-Ner et al. (2004) or Brandstätter and Güth (2002), who find a negative relationship between giving in a Dictator Game and performance on cognitive tests. By contrast, Benjamin et al. (2013) find that school test scores do not affect the Dictator's giving and, somewhat related, Hauge et al. (2009) argue that the effect of cognitive load on giving “*is small if at all existing*…” (p. 15)^1^.

Prompted by the paucity of clear-cut evidence in the field, this paper aims at shedding light on the relation between pro-social attitudes and cognitive abilities. To this aim, we borrow the design and the experimental evidence of Di Cagno et al. (2013), who set up a complex experimental design to estimate subjects' social preferences *over utilities*, where the latter include others' risk and distributional concerns. In their protocol, social preferences are elicited by submitting 98 subjects to a sequence of 24 dictatorial “situations,” which differ upon the distributional characteristics of the Dictator's choice sets. In *Standard Dictator* situations, reducing the Dictator's monetary payoff yields an increase of that to the Recipient, as it is usually the case in the Dictator Game (e.g., Forsythe et al., 1994; Andreoni and Miller, 2002). *Efficient Dictator* situations are such that the Dictators' and Recipients' monetary payoffs move in the same direction. Thus, whenever the Dictator increases or decreases her own payoff, the Recipient's payoff increases or decreases, as well. Di Cagno et al. (2013) complete the puzzle by considering situations in which either the Dictator's or the Recipient's payoff is held constant over the entire Dictator's choice set, so that *Only Recipient*s or *Only Dictators* are affected by the Dictators' decision. This novel design allows us to explore a wider spectrum of distributional concerns than what has been usually studied in standard Dictator games.

Cognitive abilities are elicited in our experiment by way of the Cognitive Reflection Test (CRT, Frederick, 2005). The CRT is a 3-item task designed to measure the tendency to override an intuitive and spontaneous response alternative that is incorrect and to engage in further reflection that leads to the correct response.

Following Cueva et al. (2015), we partition our Dictator pool into 3 subgroups (“types”): those characterized by high cognitive reflection (*reflective* subjects: 2 right answers or more in the CRT, about 40% of our subject pool), high cognitive impulsiveness (*impulsive* subjects: 2 intuitive, spontaneous incorrect answers or more in the CRT, about 50% of our subject pool) and the remainder (*others*: about 10%). Our evidence shows that reflective Dictators are more selfish whenever they can increase their own payoffs, even at the cost of the Recipients' (i.e., *Standard Dictator* and *Dictators Only* situations). By contrast, reflective Dictators are more altruistic in situations where their payoffs are not affected in terms of giving (i.e., *Recipients Only* situations). Finally, no significant differences are observed in the *Efficient Dictator* situations.

In order to test the robustness of our findings, we also study the Dictators' decisions through the lens of the classic model of social preferences by Fehr and Schmidt (1999), and provide a structural estimation of our Dictators' *envy* (i.e., aversion to inequality experienced from an disadvantaged position) and *guilt* (i.e., aversion to inequality experienced from an advantaged position). Our estimates are conditioned on Dictators' cognitive types to show that that *inequality aversion*—i.e., positive envy and guilt- is *typical of impulsive Dictators*, especially in standard dictatorial situations. By contrast, *reflective Dictators are characterized by negligible social concerns*, with the exception of an *unconditional altruistic attitude*—i.e., negative envy and positive guilt- in situations where the Dictator's payoff is held constant^2^. These findings are robust to a different and much less demanding statistical specification by which the relative shares of the pie the Dictator allocates to herself and to the Recipient are regressed against our *CRT* partition dummies.

## Materials and methods

### Participants

A total of 196 students were recruited among the undergraduate population of LUISS Guido Carli in Rome using the ORSEE recruiting system (Greiner, 2004). At the beginning of each experimental session, participants were randomly selected to play either as Dictators or Recipients in a sequence of distributional situations, and maintain their role throughout. Our Dictator pool consists of 98 subjects (51 female; mean age = 22.6 years; *SD* = 2.4 years). The experiment was run in Italian, was approved by the Ethics Committee of LUISS Guido Carli and conformed to the relevant regulatory standards^3^.

### Task

Subjects are matched in pairs for a total of 24 rounds. In each round, Dictators see two colored bar graphs with monetary amounts displayed on the top of each bar, and one slider at the bottom of the screen^4^. The Dictator must choose a specific *allocation*, γ ∈ {0, 0.01, 0.02,…, 1} by moving the slider. An allocation consists of a pair of monetary prizes, (*x_D_* (γ), *x_R_* (γ)), with

$$x_{i}\left(\gamma \right)=\left(1-\gamma \right)x_{i}^{0}+\gamma x_{i}^{1},i\in \left\{D,R\right\},$$

where *x_i_* (γ) denotes the monetary prize player *i* receives if the Dictator chooses allocation *γ*, and is calculated as the convex linear combination between the payoffs *x*^0^_*i*_ and *x*^1^_*i*_, coordinates of the endpoints of the segment which corresponds to the distributional situation set the Dictator is facing. The latter can move γ along the segment as many times as they want, until she confirms her choice by pressing the “OK” button.

We denote by $\theta =\frac{x_{D}^{1}-x_{D}^{0}}{x_{R}^{1}-x_{R}^{0}},\;x_{R}^{1}\neq x_{R}^{0},$ the exchange rate between the Dictator's and the Recipient's payoff across the Dictator's choice set. Across all 24 situations, parameters vary to ensure that Dictators face problems of different distributional characteristics. In 10 situations, Dictators play a *Standard Dictator* situation, where θ < 0. These situations are such that, by increasing her own payoff, the Dictator lowers that of the Recipient. The Dictator can increase or decrease both players' payoff simultaneously -at varying exchange rates- in what we call *Efficient Dictator* situations. Dictators face 5 decisions of this type of game, where θ > 0. In 4 situations, θ = 0 what implies that the Dictator's prize stays constant, while the Recipient's payoff varies. Finally, 5 situations are such that θ = ∞, so that the Recipient's payoff is held constant and only the Dictator's payoffs is affected^5^.

We note that the experimental design makes it possible to measure a wider variety of the Dictators' distributional concerns, since Standard Dictator situations only cover the case of θ < 0, both in case of the “classic” Dictator Game (Forsythe et al., 1994) where θ = − 1, and also in the case of the so-called “generalized” Dictator Game employed by Andreoni and Miller (2002), where θ < 0 will measure the “cost of giving.”

At the end of the phase, one round is picked at random and both the Dictator and the Recipient are paid according to the Dictator's choice in that round.

#### CRT

During the debriefing phase, among other questions, subjects are asked to complete the CRT, the well-known three-item task proposed by Frederick (2005). These questions are meant to measure the tendency to override an intuitive response alternative that is incorrect and to engage in further reflection that leads to the correct response.

**Table:** 

CRT_1_.	A bat and a ball cost $1.10. The bat costs $1.00 more than the ball. How much does the ball cost? ___ cents. **Intuitive (Incorrect) Answer: 10 / Correct Answer: 5**.
CRT_2_.	If it takes 5 machines 5 minutes to make 5 widgets, how long would it take 100 machines to make 100 widgets? _____ minutes. **Intuitive (Incorrect) Answer: 100 / Correct Answer: 5**.
CRT_3_.	In a lake, there is a patch of lily pads. Every day, the patch doubles in size. If it takes 48 days for the patch to cover the entire lake, how long would it take for the patch to cover half of the lake?_____ days. **Intuitive (Incorrect) Answer: 24 / Correct Answer: 47**.

As Frederick (2005) points out, the beauty of the test lies on the fact that “…*The three items on the CRT are ‘easy’ in the sense that their solution is easily understood when explained, yet reaching the correct answer often requires the suppression of an erroneous answer that springs ‘impulsively’ to mind*.” (p. 27)^6^.

Following Frederick (2005), it is standard practice to use the CRT to build an index -an integer from 0 to 3- by simply counting the number of correct answers. This score is then used to partition the subject pool depending on subjects' individual degree of “cognitive reflection.” Instances of this approach are the papers of Brañas-Garza et al. (2012) and Grimm and Mengel (2012), who split subjects into two groups, depending on whether their CRT score equals 0 or 3, respectively^7^.

By this methodology, “impulsive” subjects are defined as those who perform poorly in the CRT, regardless of whether they have selected the intuitive answers. To correct for this potential loss of information, Cueva et al. (2015) introduce a “dual” measure associated with the CRT, not only along the “reflective” dimension, but also along the “impulsive” one, as follows:

$$iCRT=1\left(CRT_{1}=10\right)+1\left(CRT_{2}=100\right)+1\left(CRT_{3}=24\right),$$

where 1(.) is an indicator function that takes the value 1 if condition (.) is satisfied, and 0 otherwise. In words, the *iCRT* index is meant to capture that *the inability to suppress the erroneous answer*, which provides as important information as the CRT in characterizing our subject pool. In what follows, we shall use this approach to define *reflective subjects* as those who guess two or more correct answers in the CRT. *Impulsive subjects* are defined as those who guess two or more correct answers in the *i*CRT. There is a residual subgroup (*Others*) for the remainder^8^.

### Testable hypothesis

The chief question we want to investigate is whether subjects characterized by a different degree of cognitive (ir)reflection exhibit different distributional concerns, depending on the nature of the dictatorial situation. The null hypothesis we want to test can be framed as follows:

H_0_: *In all situations, the behavior of reflective and impulsive Dictators is the same*.

Put it differently, our aim is to test whether (and how) pro-social behavior can be related to cognitive abilities, and if (and how) this relation varies across the four distributional situations.

## Results

### Descriptive statistics

We begin by summarizing our data with regard to CRT performance. Table 1 reports our Dictators' *CRT* and *i*CRT scores and partitions our dataset by relying on our definition of *reflective* (*CRT* ≥ 2) and *impulsive* (*i*CRT ≥ 2) Dictators. We observe that 49 of our 96 Dictators (51%) are *impulsive*, whereas 40 Dictators (41.7%) are *reflective*. These two categories represent roughly 93% of our sample, with only 7 subjects being categorized as *other* Dictators^9^.

**Table 1:** **CRT, iCRT, and Dictators' types**.

	**Score**				**Dictators' type**		
	**0**	**1**	**2**	**3**	***Reflective***	***Impulsive***	***Others***
CRT	35	21	18	22	40 (41.7%)	49 (51.0%)	7 (7.3%)
	(36.5%)	(21.9%)	(18.8%)	(22.9%)			
*i*CRT	25	22	27	22			
	(26.0%)	(22.9%)	(28.1%)	(22.9%)			

We now move to our behavioral data. We define σ (γ) and ρ (γ) as two *ad-hoc* proxies for “pro-social” and “selfish” behavior, respectively. More specifically, σ (γ) is calculated as the share of the Recipient's available pie the Dictator allocates to the Recipient. The value of ρ (γ) indicates the share of the Dictator's available pie the Dictator allocates to herself.

(1) $$\sigma (\gamma )=\frac{x_{R}\left(\gamma \right)-\text{min}\{x_{R}^{0},x_{R}^{1}\}}{abs\;(x_{R}^{0}-x_{R}^{1})}$$

(2) $$\rho (\gamma )=\frac{x_{D}\left(\gamma \right)-\text{min}\{x_{D}^{0},x_{D}^{1}\}}{abs\;(x_{D}^{0}-x_{D}^{1})}$$

According to Equation (1), σ (γ) = 1 (σ (γ) = 0) if the Dictator gives the Recipient the maximum (minimum) prize available to the Recipient. By the same token, by Equation (2), ρ (γ) = 1 (ρ (γ) = 0) if the Dictator gives herself the maximum (minimum) prize available.

Both variables σ (γ) and ρ (γ) are conditional on the specific round choice set; i.e., the type of game (θ). In *Standard Dictator* situations (θ <0), the share of the pie that Dictators decide to give away corresponds to what they do not keep for themselves; i.e., σ (γ) = 1 − ρ (γ). Since the Dictator cannot affect her own (the Recipient's) payoff in situations where θ = 0 (θ = ∞), the values of ρ (γ) (σ (γ)), in these cases, are not defined. Finally, in *Efficient Dictator* situations (θ >0) we have σ (γ) = ρ (γ) because Dictators increase or decrease their own payoff if and only if they increase or decrease the payoff to the Recipient.

In Table 2 we report the correlation coefficients for the relationship between the Dictator's behavior in each type of game (θ) and cognitive abilities (i.e., the score in the CRT and the *i*CRT). The average values of σ (γ) and ρ (γ) across the different situations are also reported in Table 2 (standard deviation within brackets), for our partition of reflective and impulsive Dictators^10^. Mann-Whitney non-parametric statistics are used to test our null hypothesis of no difference in behavior between that reflective and impulsive Dictators.

**Table 2:** ***CRT* and distributional concerns disaggregated for each Game type (θ)**.

**Type of Game**	**Measure**	***Correlation***		***Reflective***	***Impulsive***	***Mann-Whitney***
		***CRT***	***iCRT***	**(1)**	**(2)**	**(1) = (2)**
Standard Dictator (θ < 0)	σ (γ)	−0.091^***^	0.105^***^	0.079 (0.22)	0.115 (0.24)	−2.10^**^
Only Recipients (θ = 0)	σ (γ)	0.142^***^	−0.111^**^	0.742 (0.41)	0.640 (0.42)	2.06^**^
Efficient Dictators (θ > 0)	σ (γ) = ρ (γ)	0.037	−0.002	0.954 (0.18)	0.952 (0.18)	−0.09
Only Dictators (θ = ∞)	ρ (γ)	0.099^**^	−0.080^*^	0.994 (0.04)	0.977 (0.11)	1.67^*^
N		96	96	40	49	

The first two columns report the correlation coefficients for the relationship between the Dictator's behavior and CRT and iCRT scores, respectively. The last column reports the results of Mann-Whitney non-parametric tests where the null hypothesis states that means are constant across CRT types. Significance at the

*** *1%*,

** *5%*,

* *10% level*.

The results in Table 2 indicate that a higher score in the CRT will result in less generous behavior in the Standard Dictator situations (the opposite being true for the *i*CRT). This, in turn, implies that reflective subjects give less than impulsive subjects in the Standard Dictator situations (0.079 vs. 0.115, MW-test: 2.10, *p* < 0.036), in line with the empirical evidence in Ben-Ner et al. (2004) or Brandstätter and Güth (2002)^11^. In sharp contrast, reflective subjects give significantly more than impulsive when the Dictator's payoff is held constant and only Recipients' payoffs is affected (0.742 vs. 0.640, MW-test: 2. 60, *p* < 0.039). A different pattern is observed in situations where taking does not affect the Recipient's payoff (θ = ∞). In that case, reflective subjects tend to be more selfish, although differences are only significant at the 10% level (0.994 vs. 0.977, *U*-test: 1.67, *p* < 0.095). Finally, we do not detect significant differences in aggregate behavior in Efficient Dictator situations (0.954 vs. 0.953, MW-test: −0.48, *p* = 0.631). Thus, when Dictators' and Recipients' incentives are aligned, both reflective and impulsive Dictators opt for the most efficient allocation in the vast majority of cases.

In Section Robustness Check I. Random-effect Tobit Regressions, we use random-effect tobit regressions in which σ (γ) and ρ (γ) are regressed against our CRT partition dummies to show that our findings are robust across different econometric specifications. We extend our modeling strategy in Section Robustness Check II. Structural Estimation of Fehr and Schmidt (1999), where we follow Cabrales et al. (2010) to estimate the model of social preferences proposed by Fehr and Schmidt (1999).

### Robustness check I. random-effect tobit regressions

Table 3 reports our estimates after running random-effect tobit regressions for σ (γ) and ρ (γ) in each type of game. The set of regressors includes the Dictator's type (reflective, impulsive, other), the round in which the decision is made, and the Dictator's gender (a dummy variable positive for female)^12^. The reported standard errors (in parenthesis) are clustered by subject.

**Table 3:** **Random-effect tobit regression for σγ and ργ in each type of game**.

	**Standard dictator (θ <0)**		**Only recipient (θ = 0)**		**Efficient dictator (θ > 0)**		**Only dictator (θ = ∞)**	
	**σ (γ)**	**σ (γ)**	**σ (γ)**	**σ (γ)**	**σ (γ)**	**σ (γ)**	**ρ (γ)**	**ρ (γ)**
*Reflecitve (R)*	−0.719^***^	−0.651^***^	1.759^***^	1.929^***^	3.103^***^	2.926^***^	2.296^***^	2.137^***^
	(0.10)	(0.10)	(0.25)	(0.27)	(0.53)	(0.52)	(0.33)	(0.30)
*Impulsive (I)*	−0.454^***^	−0.333^***^	1.154^***^	1.462^***^	3.003^***^	2.686^***^	1.889^***^	1.602^***^
	(0.09)	(0.10)	(0.22)	(0.26)	(0.51)	(0.51)	(0.25)	(0.22)
*Others*	−0.535^***^	−0.445^***^	1.310^***^	1.614^***^	2.469^***^	2.148^***^	2.211^***^	1.980^***^
	(0.15)	(0.15)	(0.40)	(0.42)	(0.59)	(0.59)	(0.43)	(0.41)
*Round*	−0.003	−0.003	−0.002	−0.001	0.001	0.004	0.017	0.016
	(0.01)	(0.01)	(0.01)	(0.01)	(0.02)	(0.02)	(0.01)	(0.01)
*Female*		−0.179^**^		−0.507^**^		0.448		0.476^***^
		(0.07)		(0.20)		(0.29)		(0.18)
*Wald test (R) = (I)*	3.48^***^	3.99^***^	2.87^***^	2.19^**^	0.35	0.79	2.15^**^	2.65^***^
Observations	960	960	480	480	384	384	480	480

The Wald test after each regression is used to test the behavioral hypothesis that reflective and impulsive subjects behave in the same manner. Significance at the

*** *1%*,

** *5%*,

* *10% level*.

As Table 3 shows, reflective subjects give less than impulsive in the Standard Dictator situations (*W* = 3.48, *p* < 0.001), even after controlling for round and gender (*W* = 3.99, *p* < 0.001). The Wald tests confirm the difference in pro-social behavior, with reflective subjects being more altruistic in the *Only Recipient* situations and more selfish in the *Only Dictator* ones. No differences are observed in the *Efficient Dictator* situations.

Overall, these results confirm that (i) reflective Dictators are more selfish when they are allowed to increase their own payoff, regardless of whether or not Recipients pay a cost for it, and that (ii) reflective Dictators are more generous when they are not harmed, in absolute terms, by giving more^13^.

### Robustness check II. structural estimation of Fehr and Schmidt (1999)

This section posits that Dictators' behavior follows the classic social preference model by Fehr and Schmidt (1999). According to this model, the Dictator's utility, *u*(.), does not only depend on her own monetary payoff *x_D_* (γ), but also on that of the Recipient, *x_R_* (γ), as follows:

(3) $$\begin{array}{l} u(x_{D}\left(\gamma \right),x_{R}\left(\gamma \right))=x_{D}\left(\gamma \right)-\;\alpha \text{max}\left\{{\text{x}}_{\text{R}}\left(\gamma \right){\text{-x}}_{\text{D}}\left(\gamma \right),\text{0}\right\}\\ \;-\beta \text{max}\left\{{\text{x}}_{\text{D}}\left(\gamma \right){\text{-x}}_{\text{R}}\left(\gamma \right),\text{0}\right\}, \end{array}$$

where α and β measure the Dictator's *envy* (i.e., aversion to inequality when receiving less than the Recipient) and *guilt* (i.e., aversion to inequality when receiving more than the Recipient), respectively.

**Figure 1:**
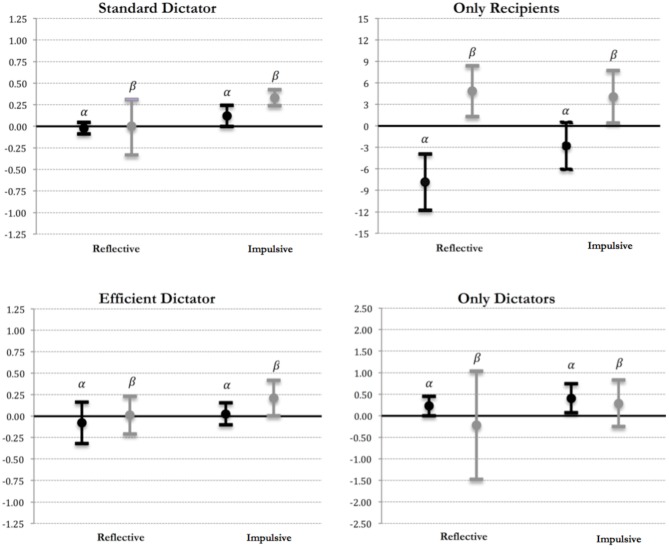
**Estimates of Fehr and Schmidt (1999) in each type of game**. Point estimates and confidence intervals for *envy* (α) and *guilt* (β) are reported in black and gray, respectively.

In what follows, we estimate by maximum likelihood the unconstrained coefficients of Equation (3) by considering a multinomial logit model, clustered at the subject level (see Appendix A in Supplementary Material). Figure 1 provides a graphic display of our point estimates of α and β for reflective and impulsive subjects, together with the 95% confidence interval, for each game type, θ^14^.

As Figure 1 shows, our estimates for α and β for reflective Dictators are not significantly different from zero in the standard Dictator situations (using the Wald test, the null hypothesis *H*_0_: α = β = 0 cannot be rejected for reflective subjects at any common significance level, with χ^2^_2_ = 0.70 and *p* = 0.706). This is in contrast with evidence for *impulsive* subjects (χ^2^_2_ = 49.30, *p* < 0.001). These findings suggest that in Standard Dictator situations *inequality aversion is just typical of impulsive subjects*. By contrast, reflective dictators are instead characterized by “selfish” social preferences, in that both α and β are not statistically different from 0.

We also find evidence for inequality aversion when the Dictators' payoff is held constant, as both α and β are statistically different from 0 for *reflective* and *impulsive* subjects in the *Only Recipients* situations (the null hypothesis *H*_0_: α = β = 0 is rejected for both type of subjects, with *p*-values being 0.0003 and 0.089, respectively). In addition, we see that α is significantly smaller for reflective subjects than for impulsive subjects (χ^2^_1_ = 4.57, *p* = 0.033), with α being significantly smaller than β for both type of subjects (in both cases, *p* < 0.016). These findings indicate that, although both reflective and impulsive subjects care about inequality, impulsive Dictators are more envious. As a result, reflective Dictators tend to be more generous than impulsive Dictators when choices do not affect their earnings. This evidence is in line with our analysis in Table 2, as well as our finding for the Efficient Dictator situations, in which we observe no significant differences between reflective and impulsive Dictators with regard to *both* the values of α (*p* = 0.427) and β (*p* = 0.235). When we analyze the situations where only the Dictator's payoff varies, our estimates of α and β for reflective and impulsive subjects are not significantly different from each other. Interestingly, the estimate of α is positive and significant both cases, what indicates that Dictators account for envy (but not for guilt) when the Recipient's payoff is held constant.

## Discussion

This paper, and the experimental evidence reported herein, lies between two conflicting views that are well established in the current economic debate. One of them identifies “rationality” with “selfishness” and predicts that highly cognitive subjects should exhibit less pronounced distributional concerns. The other one, which appeals to the “too-smart-to-be-selfish” view, highlights the long-term benefits of altruism (in terms of positive reciprocity, social efficiency and the like) and calls, consequently, for a positive relation between cognitive abilities and altruism. Both views find some empirical support in the -relatively limited- experimental literature on these matters (take, for example, Brandstätter and Güth, 2002 for the former and Millet and Dewitte, 2007 for the latter). Our aim is to present further evidence in order to contribute to this lively and intriguing debate.

We consider four different versions of the Dictator Game, which differ in the way in which Dictators' and Recipients' payoffs are affected by the Dictators' choices. To elicit cognitive abilities, we rely on our CRT partition into three “cognitive types.” This, in turn, allows us to tease apart the behavior of r*eflective* and *impulsive* Dictators, by relying not only on the “reflective” dimension of the test, but also on the “impulsive” one.

Our main findings suggest that *reflective* Dictators are more selfish than *impulsive* Dictators in Standard Dictator situations, in which Dictators increase their own payoffs at the cost of the Recipient. By contrast, reflective Dictators are more generous when the “opportunity cost of giving” is comparatively low (i.e., when being more generous does not affect own payoffs). Using a parametric approach, we indeed confirm that “inequality aversion” (i.e., positive α and β) best describes the behavior of impulsive Dictators in Standard Dictator situations, in which reflective Dictators show little or no distributional concerns. By contrast, in situations where the Dictator's payoff is held constant, reflective subjects give significantly more (i.e., they are significantly more guilty and less envious than their impulsive counterparts). Overall, these findings complement the recent work of Hauge et al. (2009), who attempt to see whether the Dictators' natural instinct is to be selfish and social preferences require some cognitive reasoning. To that purpose, the authors investigate whether introducing *cognitive load* (more specifically, asking subjects to remember complicated numbers) leads to more selfish behavior. Our results suggest that subjects who are impulsive subjects are more inequity averse, whereas reflective subjects are more selfish, except when there is nothing in it for them.

Along these lines, there is a stand of the literature in altruism and evolution that investigates the importance of “intelligence” on behavior. In this literature, the so-called *Neo-Darwinian* theory (see, e.g., Dawkins, 1976) suggests that altruism may be detrimental as it reduces the one's fitness while enhancing the fitness of others. Simon (1993), however, argues that “intelligent altruists,” although less altruistic than the unintelligent altruists, “will be fitter than both unintelligent altruists and selfish individuals” given that human beings are characterized by bounded rationality and may learn from other individuals what is good for them (i.e., social influence may grease the wheels for altruistic behavior)^15^. Because the CRT has a substantial correlation with cognitive ability and intelligence (see Frederick, 2005; Kahneman, 2011; Toplak et al., 2011), the CRT measure nicely contributes to this debate by suggesting that the effect of cognitive reflection on pro-social behavior is critically linked to whether (and how) Dictators need to pay the cost of their supposed “generosity.” We acknowledge, however, that the CRT is a particular way of measuring intelligence and it has some distinctive features (Toplak et al. 2011), therefore it may be worth complementing our findings with those applying alternative measures of cognitive ability, such as strategic sophistication (Coricelli and Nagel, 2009).

### Conflict of interest statement

The authors declare that the research was conducted in the absence of any commercial or financial relationships that could be construed as a potential conflict of interest.

## Footnotes

^1^Hauge et al. (2009) attempt to see whether the Dictator's natural instinct is to be selfish and social preferences require some cognitive reasoning. To that purpose, the authors ask subjects to memorize numbers of 7 digits, some of which are easy (hard) to remember, e.g., 1111111 or 1234567 (9325867 or 7591802). Hauge et al. (2009) fail to find a clear relationship between attitudes to give in different pro-social tasks (e.g., charitable giving, Dictator Games, etc…) and “cognitive load” (i.e., the ability to remember more complex numbers). Benjamin et al. (2013) manipulates also the cognitive resources for evaluating choices to investigate the effect on risk aversion and discounting. Strategic thinking and the degree of bounded rationality (as measured by the subject's performance in a guessing game) is related to behavior in the dictator game in Arruñada et al. (2015) and Dittrich and Leipold (2014).

^2^For a similar approach, see Corgnet et al. (2015).

^3^Appendix B in Supplementary Material contains a translated version of the instructions for the phase in which pro social behavior was elicited. This includes a screenshot for the Dictators' decision, detailed in Section Task.

^4^Figure B1 in Appendix B (Supplementary Material) reports a screenshot of the user interface.

^5^The details of specific distributional situations and the between-subject randomization are reported in Appendix C (Supplementary Material).

^6^One of the characteristic features of the test, besides its simplicity, is indeed its relationship with System 1 and System 2 cognitive functioning. “*System 1 thinking refers to our intuitive system, which is typically fast, automatic, effortless … whereas System 2 refers to reasoning that is slower, effortful, logical …*” (see Kahneman, 2011).

^7^Brañas-Garza et al. (2012) consider the 0, 1 cutoff so that subjects who scored 0 in the CRT are in a different category that those having 1, 2 or 3 correct answers. The cutoff in Grimm and Mengel (2012) is 2, 3; i.e., subjects who scored 3 in the CRT are in a different category that those scoring 0, 1 or 2.

^8^Somehow related, Noussair et al. (2014) define an Enhanced CRT (ECRT) that punishes intuitive, spontaneous incorrect answers more severely than all other incorrect answers. Thus, an erroneous response counts -1 in their index if it was the spontaneous, intuitive answer, but it counts 0 otherwise.

^9^The interested reader can see the histograms for the responses to the CRT in Figure A1 in Appendix A (Supplementary Material). This includes Dictators' CRT and iCRT scores disaggregated for gender (Table A1).

^10^Recall that a total of 7 observations correspond to *O*ther Dictators. For them, the average value (standard deviation) of σ (γ) is 0.120 (0.28) in the Standard Dictator game, 0.649 (0.44) in the Only Recipients game, and 0.892 (0.31) in the Only Dictators game. In the Only Dictators game, the average value (standard deviation) of ρ (γ) is 0.996 (0.02). Figure 2A in Appendix A (Supplementary Material) reports the distribution of σ (γ) and ρ (γ) for each.

^11^Chen et al. (2013) find in their study that better performance in the SAT is associated with more generosity, although subjects with higher Grade Point Averages (GPA) are more selfish in the dictator decisions. As pointed out by one of the referees, Chen et al. (2013) conjecture that grade point average, unlike SAT scores, would be closer to an incentivized test of cognitive ability, as it is the CRT. Along these lines, our findings nicely corroborate the grade point average result of Chen et al. (2013) for the GPA scores, which are relatively unaffected by the intrinsic motivation of the subjects. We are thankful to the referee for this comment.

^12^Note that we do not include any constant, but the three different dummies for the Dictator's type.

^13^Our findings are robust if we consider instead the score in the CRT as explanatory variable. See Table A3 in Appendix A (Supplementary Material).

^14^Appendix A (Supplementary Material), together with a more detailed description of the structural estimation strategy, reports in Table A4 our estimated coefficients for all categories, including *other* Dictators, which are omitted in Figure 1 for the sake of simplicity.
