# Supplementary material for: Social preferences and cognitive reflection: evidence from a dictator game experiment
Source: Front Behav Neurosci. 2015 Jun 19;9:146. doi: 10.3389/fnbeh.2015.00146 (PMC4473592; doi:10.3389/fnbeh.2015.00146)
Supplement: Supplementary file 1 [file Table1.PDF]

Social Preferences and Cognitive Reflection: Evidence from Dictator Game Experiment

[Supplementary Material]

Giovanni Ponti[•]
Universidad de Alicante and
LUISS Guido Carli Roma

Ismael Rodriguez-Lara
Middlesex University London and
LUISS Guido Carli Roma

[•] Corresponding author. Dipartimento di Economia e Finanza, LUISS Guido Carli. Viale Romania, 32. 00185 Roma. E-mail: gponti@luiss.it.

Appendix A. Additional Empirical Evidence

Figure A1 reports the distributions of the responses in the CRT for our subject pool.

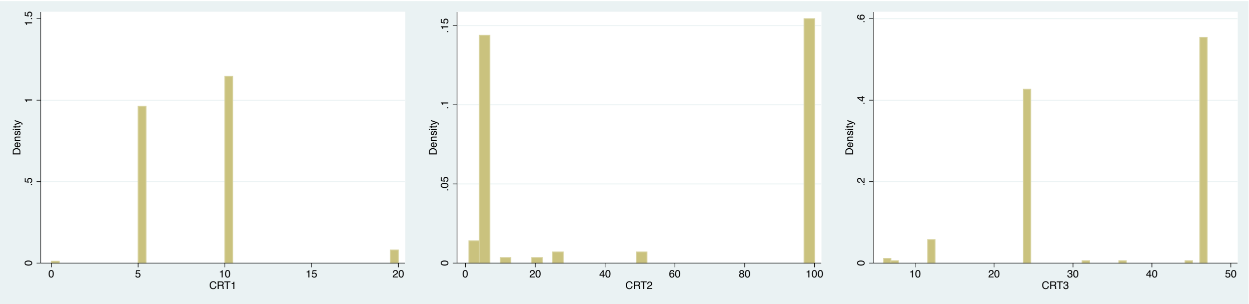

Figure A1. Histogram for the responses to the CRT

Recall that the correct (intuitive/spontaneous incorrect) answers to the CRT test are 5, 5, and 47 (10, 100 and 24), respectively. Also notice that these intuitive incorrect answers correspond to the modal choices in our dataset, as well as in the relevant references (see, e.g., Frederick, 2005 or Gill and Prowse, 2014).

Table A1 reports our Dictators' *CRT* and *iCRT* scores disaggregated for gender in Panel *a)* and *b)*, respectively. Panel *c)* partitions our dataset by relying on the definition of *Reflective* (CRT ≥ 2) and *Irreflective* (*i*CRT ≥ 2) Dictators we put forward in Section 2.

CRT	Male	Female	Total
**0**	11	24	35 (36.5%)
**1**	9	12	21 (21.9%)
**2**	9	9	18 (18.8%)
**3**	16	6	22 (22.9%)
**TOT**	45	51	96

a)

*i*CRT	Male	Female	Total
**0**	18	7	25 (26.0%)
**1**	10	12	22 (22.9%)
**2**	12	15	27 (28.1%)
**3**	5	17	22 (22.9%)
**TOT**	45	51	96

b)

CRT-partition	Male	Female	Total
Reflective	25	15	40 (41.7%)
Irreflective	17	32	49 (51.0%)
Others	3	4	7 (7.3%)
**TOT**	45	51	96

c)

Table A1. CRT, *i*CRT, Dictators' types and gender

Consistent with the literature, male show a higher average score in CRT than female (1.67 *vs.* 1.00, respectively), while the opposite holds for *iCRT* (1.09 *vs.* 1.82). The Pearson's correlation coefficient between the Dictator's gender and the score in the CRT and the *i*CRT are highly significant (*p*-values < 0.0001). Panel *c)* shows that 49 of our Dictators are *irreflective* (65% of these subjects are female), whereas 40 Dictators are *reflective* (62.5% of these subjects are male). By contrast, the residual group, *Others,* distributes almost equally across genders. These results are in line with the findings of Cueva *et al.* (2014), from which we borrow the methodology of this specific CRT partition and deals with a much larger database (nearly 1.200 subjects).

Figure A2 reports frequency data on the mean values of $\sigma(\gamma)$ and $\rho(\gamma)$ for each distributional problem and

each type of Dictator separately. As Figure A2, Panel *a)* shows, values of σ are smaller for reflective subjects in the Standard Dictator situations, which suggests that they are less generous that impulsive Dictators in this situation. In line with our description of the data, we also observe a larger σ for reflective Dictators in the Only Recipient situations, and a larger ρ in the Only Dictator situations (see Panel *b*).

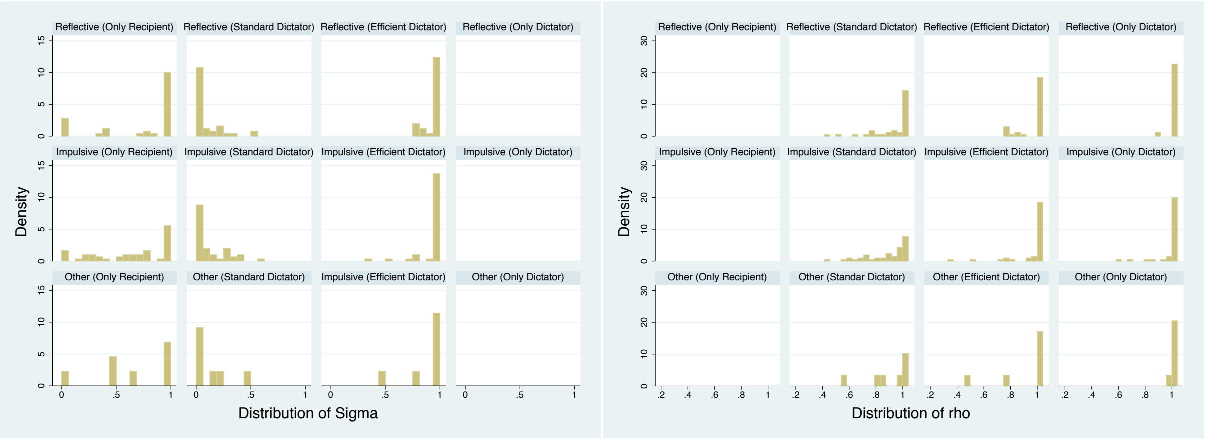

a) *b)*

Figure A2. Distributions of sigma and rho across CRT types and dictatorial situations.

Table A2 reports correlation coefficients between σ(γ) (ρ(γ)) and CRT scores and partitions, by dictatorial situation.

	Standard Dictator ($\theta < 0$)	Only Recipient ($\theta = 0$)	Efficient Dictator ($\theta > 0$)	Only Dictator ($\theta = \infty$)
	$\sigma(\gamma)$	$\sigma(\gamma)$	$\sigma(\gamma)$	$\rho(\gamma)$
CRT	-0.09***	0.14***	0.037	0.1**
iCRT	0.1***	-0.11**	-0.002	0.08*
Reflective	-0.08**	0.12***	0.023	0.09**
Impulsive	0.06**	-0.1**	0.019	-0.11**
Others	0.02	-0.02	-0.081	0.04
Observations	960	480	384	480

Tab. A2. Beta correlation coefficients between $\sigma(\gamma)$ ($\rho(\gamma)$) and CRT scores and partitions, by dictatorial situation. Significance at the *** =1%, **=5%, *=10% level.

Consistently with our results in Section 3, Reflective subjects give less (more) than impulsive in Standard and Only Dictator (Only Recipients) situations. No significant correlations are found in the case of the Efficient Dictator situations and (more interestingly) for the residual group (Others), who show a behavioral pattern different than the other two groups in all distributional situations. This evidence supports our empirical strategy of considering two relevant dimensions, *reflectiveness vs. impulsiveness,* instead of only one: subjects who are neither reflective nor impulsive (7% in our sample, but 20% in Cueva *et al.,* 2014) have a different socio-demographic composition (take, e.g., the absence of a gender bias) and a different aggregate behavior in the experiment.

We also calculate correlation coefficients between individual mean $\sigma(\gamma)$ ($\rho(\gamma)$) across different situation types.[1] In this respect, we find that

1. For Reflective subjects, the correlations between average $\sigma(\gamma)$ across situations are never significant.

[1] Full sets of correlations are not reported here, but available upon request.

By contrast, we find positive correlations (significant at 5% confidence level) between mean $\rho(\gamma)$ in Only Dictators situations and mean $\rho(\gamma)$ in Standard and Efficient Dictator situations. In other words, among Reflective Dictators, those who keep more for themselves in Only Dictator, so behave also in Standard and Efficient Dictator situations. As we already know from the evidence in Section 3, for reflective Dictators, their behavior follows a rather different pattern in Only Recipient situations, which, indeed, is not correlated with the behavior of reflective Dictators in the other distributional situations.

2. As for impulsive Dictators, all $\rho(\gamma)$ are positively correlated (at least, at 10% significance), while, in the case of $\sigma(\gamma)$, we find a positive (negative) correlation between Efficient Dictator and Only Recipient (Standard Dictator) situations. In other words, those Impulsive who give more in Efficient Dictator, also give more (less) in Only Recipient (Standard Dictator) situations, respectively. This is a reflection of the elicited *inequity aversion* we observe in the structural estimates of Table A5.
3. No significant correlations are found in case of the residual group, *others*. This is a further sign that the latter is characterized by behavioral patterns qualitative distinguishable from those of the other two.

Table A3 complements the content of Table 3 by reporting the estimated coefficients of random-effects obit regressions in which the link between $\sigma(\gamma)$ and $\rho(\gamma)$ and cognitive reflection, *ceteris paribus,* is not measured by our type partition –as in Table 3- but simply by the CRT score, directly.

	Standard Dictator ($\theta < 0$)		Only Recipient ($\theta = 0$)		Efficient Dictator ($\theta > 0$)		Only Dictator ($\theta = \infty$)	
	$\sigma(\gamma)$	$\sigma(\gamma)$	$\sigma(\gamma)$	$\sigma(\gamma)$	$\sigma(\gamma)$	$\sigma(\gamma)$	$\rho(\gamma)$	$\rho(\gamma)$
CRT	-0.124***	-0.149***	0.295***	0.236***	0.47	0.225*	0.198**	0.266***
	(0.03)	(0.03)	(0.09)	(0.09)	(0.12)	(0.13)	(0.08)	(0.09)
Round	-0.003	-0.003	-0.002**	-0.003**	0.002	0.004	0.017	0.015
	(0.005)	(0.005)	(0.01)	(0.01)	(0.02)	(0.02)	(0.01)	(0.01)
Gender		-0.194***		-0.457***		0.528*		0.523***
		(0.07)		(0.20)		(0.30)		(0.19)
Constant	-0.415***	-0.277***	1.030***	1.335**	2.829***	2.425***	1.849***	1.517***
	(0.08)	(0.10)	(0.22)	(0.26)	(0.49)	(0.49)	(0.24)	(0.21)
Observations	960	960	480	480	384	384	480	480

Tab. A3. Random-effect Tobit regressions for $\sigma(\gamma)$ and $\rho(\gamma)$ in each type of game (standard errors in parenthesis). Significance at the *** =1%, **=5%, *=10% level.

As Table A3 shows, the CRT score has a negative (positive) effect on giving in the Standard Dictator (Only Recipients) choices. This is in line with our description of the data in Section 2.2, where we observe that reflective subjects give less (more) in Standard Dictator (Only Recipient) situations, respectively. We also observe that the score in the CRT has a positive effect on the share that Dictators allocate to themselves in the Only Dictator treatment, as we already discussed in Section 3. Consistent with our description of the data, we observe no differences in the Efficient Dictator choices with regard to the score in the CRT, although this is weakly

significant at the 10% level after controlling for gender.

Table A4 reports the full set of estimates of the structural model presented in Section 3.3, Figure 1. A standard estimation protocol is used to estimate likelihoods conditional on the model (3).[2] We estimate the probability of selecting a specific allocation, $\gamma^* \in \{0, .01, .02, \dots, 1\}$, using the following multinomial logit equation (time index omitted):

$$Prob(y_i = \gamma^* | \alpha, \beta, x) = \frac{\exp\,[\mu u(x_D(\gamma^*), x_R(\gamma^*))]}{\sum_\gamma \exp\,[\mu u(x_D(\gamma), x_R(\gamma))]}, \quad \text{(A1)}$$

where α and β are the structural parameters to be estimated, $x \equiv (x_D^0, x_D^1, x_R^0, x_R^1)$ contains the distributional parameters of the situation at stake, and $\mu>0$ is a structural "noise" parameter to allow for some error in the Dictator's evaluation of the utility associated with each allocation. When $\mu \to \infty$, this specification collapses to the deterministic choice model (3), in that the estimated probability of the utility maximizing allocation converges to one. By contrast, as $\mu \to 0$, the choice essentially becomes random.

	Standard Dictator ($\theta < 0$)		Only Recipient ($\theta = 0$)		Efficient Dictator ($\theta > 0$)		Only Dictator ($\theta = \infty$)	
	α	β	α	β	α	β	α	β
Reflective	-0.022	-0.007	-7.854***	4.864***	-0.078	0.013	0.228**	-0.220
	(0.035)	(0.162)	(2.021)	(1.811)	(0.123)	(0.112)	(0.113)	(0.640)
Impulsive	0.119*	0.330***	-2.798*	4.065**	0.027	0.211**	0.408**	0.289
	(0.063)	(0.048)	(1.678)	(1.879)	(0.065)	(0.106)	(0.170)	(0.276)
Others	-0.083	-0.464	-1.656	7.571	0.059	-0.299	0.423*	-9.777***
	(0.087)	(0.431)	(3.379)	(7.261)	(0.089)	(0.242)	(0.223)	(3.593)
μ	1.809***		0.05***		3.28***		4.199***	
	(0.2579)		(0.0121)		(0.8414)		(1.3457)	
Obs.	960		480		384		480	

*** p<0.01, ** p<0.05, * p<0.1. Robust standard errors in parentheses

Table A4. Estimates of the model of Fehr and Schdmit (1999) for each the type of game, θ. Robust standard errors within brackets.

[2] See Cabrales *et al.* (2010), for a more detailed account of the estimation strategy.

Appendix B. Experimental instructions (translated into English)

This appendix contains a translated version of the experimental instructions (originally in Italian). Below, we present the instructions of STAGE IV, including a screenshot for this stage, in which Di Cagno et al. (2014) elicit social preferences. The authors do also elicit risk preferences. The complete set of instructions is available upon request.

Welcome Screen

- Welcome to this experiment!
- This is a study to study how people make decisions. We are only interested in what your choices are.
- Your behavior will affect the amount of money you can win.
- You will make decisions in several different situations, which we will call "stages." Each decision and outcome is independent from each of your other decisions. So your decisions and outcomes in one game will not affect your outcomes in any other game.
- In some cases, you will be anonymously paired with one other person, so that your decisions may affect the **earnings** of others, just as the decisions of the other person may affect your **earnings**. In any case, the computer will ensure, in every stage, that you will be paired with a different person than in previous decisions. **You will never be paired with the same person twice.**
- Below we provide the instructions explaining what your choices are in each stage and how to use the computer.
- Please do not disturb the other participants. If you need help, raise your hand and wait in silence. We will help you as soon as possible.

STAGE IV

- As in STAGE III, a CHOOSER will be paired with a NON-CHOOSER, different from the previous pairings. This time the CHOOSER will have to choose across a set of 101 possible money allocations, consisting of a certain amount of money for the CHOOSER and a certain amount of money for her randomly matched NON-CHOOSER. **Each available allocation is identified by a number, an integer between 0 and 100, which always appear on top of the slider.**
- In this stage the screen of the CHOOSER has a slider with two bars, similar in appearance to the one you already used in stage II to bet over the others' decisions. However, this time the CHOOSER's decision on where to place the slider has different consequences: it will fix the monetary gains for the CHOOSER and the paired NON-CHOOSER for that round. As usual, at the end of this stage the computer will select one round for earning.

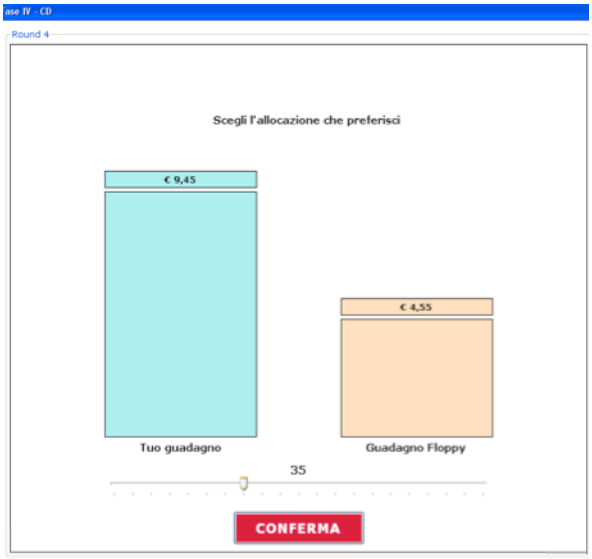

Fig. B1. Experimental user interface.

- If you are a CHOOSER, we ask you to move the slider back and forth several times before deciding where to stop and confirming your decision. You confirm your decision by clicking on the "CONFERMA" button. This will help you to get familiar with the entire set of possibilities at your disposal, before you decide on the allocation that you prefer.
- If you are a CHOOSER, you will notice that moving the slider to the right *never decreases the amount of money for your*

matched NON-CHOOSER, and typically increases the amount of money for the matched NON-CHOOSER. On the other hand, moving the slider to the right also changes the CHOOSER's amount of money. Sometimes the amount of money for the CHOOSER will also increase; sometimes the amount of money for the CHOOSER will decrease; and sometimes the amount of money for the CHOOSER will not move at all. When the amounts for the CHOOSER and NON-CHOOSER change, they might not change at the same speed. It is important that the CHOOSER is aware of all the available possibilities before confirming a decision.

- The amount for the CHOOSER is drawn as a light blue bar on the left of the screen, and the amount for the NON-CHOOSER is drawn as a pink bar on the right of the screen. The two amounts of money shown on the screen are the two amounts that would be distributed if the CHOOSER confirmed at that very moment, and this decision was selected for payment. For example, if the CHOOSER confirmed the allocation in the above example, the CHOOSER would allocate €7.32 to themself and €1.56 for the matched NON-CHOOSER.

To summarize, **your earnings in this stage are** determined by three things:

- by which round is chosen to be played out in the series of 24 rounds;
- by the set of allocations (selected by the computer) for that particular round; and
- by the decision of the CHOOSER in your matched pair.

Appendix C – Dictatorial situations: calibration.

Four sets of distributional parameters, from 1 to 4, were used between sessions (the same set for 2 out of 8 sessions). The order of decisions within each set was randomly assigned for each individual Dictator.

SET 1					SET 2					SET 3					SET4				
x_D^0	x_R^0	x_D^1	x_R^1	θ															
8	0	0	8	-1,00	4	0	0	4	-1,00	12	0	0	12	-1,00	6	0	0	6	-1,00
12	0	0	4	-3,00	11	0	0	5	-2,20	14	0	0	2	-7,00	13	0	0	1	-13,00
4	0	0	12	-0,33	5	0	0	11	-0,45	2	0	0	14	-0,14	1	0	0	13	-0,08
12	2	10	12	-0,20	11	1	10	11	-0,10	14	4	12	14	-0,20	9	2	8	10	-0,13
12	2	1	4	-5,50	9	1	2	5	-1,75	8	3	2	4	-6,00	7	1	1	5	-1,50
12	7	1	9	-5,50	14	6	2	8	-6,00	9	4	1	9	-1,60	8	5	2	7	-3,00
14	0	7	7	-1,00	12	0	6	6	-1,00	10	0	5	5	-1,00	6	0	3	3	-1,00
7	7	0	14	-1,00	6	6	0	12	-1,00	5	5	0	10	-1,00	3	3	0	6	-1,00
12	2	5	4	-3,50	10	2	6	5	-1,33	8	1	5	4	-1,00	9	3	6	5	-1,50
4	5	2	12	-0,29	5	6	2	10	-0,75	4	5	1	8	-1,00	5	6	3	9	-0,67
3	1	4	12	0,09	2	1	5	10	0,33	4	2	6	14	0,17	3	1	6	12	0,27
1	3	12	4	11,00	1	2	10	5	3,00	2	4	14	6	6,00	1	3	12	6	3,67
3	2	12	5	3,00	4	1	14	6	2,00	12	2	14	12	0,20	6	2	8	8	0,33
2	3	5	12	0,33	1	4	6	14	0,50	2	12	12	14	5,00	2	6	8	8	3,00
5	3	5	12	0,00	4	2	4	14	0,00	8	1	8	15	0,00	12	2	12	14	0,00
12	0	12	14	0,00	9	6	9	12	0,00	4	1	4	12	0,00	3	0	3	10	0,00
7,5	2	7,5	7,5	0,00	9	3	9	9	0,00	12	1	12	12	0,00	6	1	6	6	0,00
2	2	2	12	0,00	4	4	4	12	0,00	6	6	6	14	0,00	8	8	8	15	0,00
4	6	4	12	0,00	2	4	2	12	0,00	6	8	6	15	0,00	4	1	4	3	0,00
3	5	12	5	N/A	2	4	14	4	N/A	1	8	15	8	N/A	2	12	14	12	N/A
2	12	14	12	N/A	6	9	12	9	N/A	1	4	15	4	N/A	0	3	10	3	N/A
2	7,5	7,5	7,5	N/A	3	9	9	9	N/A	1	12	12	12	N/A	1	6	6	6	N/A
2	2	12	2	N/A	4	4	12	4	N/A	6	6	14	6	N/A	8	8	15	8	N/A
6	4	12	4	N/A	4	2	12	2	N/A	8	6	15	6	N/A	1	4	3	4	N/A

Referencies

Cabrales, A., Miniaci, R., Piovesan, M. and Ponti G. (2010). Social preferences and strategic uncertainty: An experiment on markets and contracts. *American Economic Review*, 100(5), 2261-2278.

Cueva, C., Iturbe-Ormaetxe, I., Mata-Pérez, E., Ponti, G., Yu, H., and Zhukova, V. (2014). *Cognitive (Ir)reflection: New Experimental Evidence*, Universidad de Alicante, mimeo.

Di Cagno, D., Harrison, G. W., Miniaci, R., and Ponti, G. (2013). *Social Preferences over Utilities*, LUISS Guido Carli Roma, mimeo.

Fehr, E. and Schmidt, K. M. (1999). A theory of fairness, competition and cooperation. *Quarterly Journal of Economics*, 114, 817-68.

Frederick, S. (2005). Cognitive Reflection and Decision Making. *Journal of Economic Perspectives*, 19(4), 25–42.

Gill, D., & Prowse, V. L. (2014). Cognitive ability, character skills, and learning to play equilibrium: A level-k analysis. *Character Skills, and Learning to Play Equilibrium: A Level-k Analysis* (June 9, 2014).
